# Longitudinal strain correlated with haemodynamics in patients with chronic thromboembolic pulmonary hypertension

**DOI:** 10.1186/1532-429X-18-S1-P299

**Published:** 2016-01-27

**Authors:** Min Liu, Tian Jing Zhang, Xiaojuan Guo, Yuanhua Yang

**Affiliations:** 1 grid.411607.5Radiology, Beijing Chaoyang Hospital of Capital Medical University, Beijing, China; 2 MR Collaborations NE Asia, Siemens Healthcare, Beijing, China; 3 grid.411607.5Respiratory Diseases Research Center, Beijing Chaoyang Hospital of Capital Medical University, Beijing, China

## Background

to determine the feasibility of the longitudinal strain assessed by Trufistrain on four-chamber images of Cardiac MRI in patients with chronic thromboembolic pulmonary hypertension and to correlate with right heart catheterization.

## Methods

After informed consent, 36 patients (age, 45.9 ± 10.1 years; male/female= 25/11, Heart rate=65 ± 8bpm) with chronic thromboembolic pulmonary hypertension (CTEPH) underwent CMRI at 3T (TimTrio, Siemens) and right heart catheterization in the same day. After Four-chamber cine images were acquired during short breath-holds with a retrospectively gated turbo FLASH gradient-echo sequence and the longitudinal strain independently measured by two radiologists on four-chamber cine images with Trufistrain (Siemens). The hemodynamics was evaluated with right heart catheterization. The feasibility of the longitudinal strain assessed by Trufistrain. The feasibility of strain evaluated with Trufistrain were assessed with A Bland-Altman plot and correlation of strain with hemodynamics was evaluated with Spearman correlation on Medicalc and SPSS

## Results

**Figure 1:**
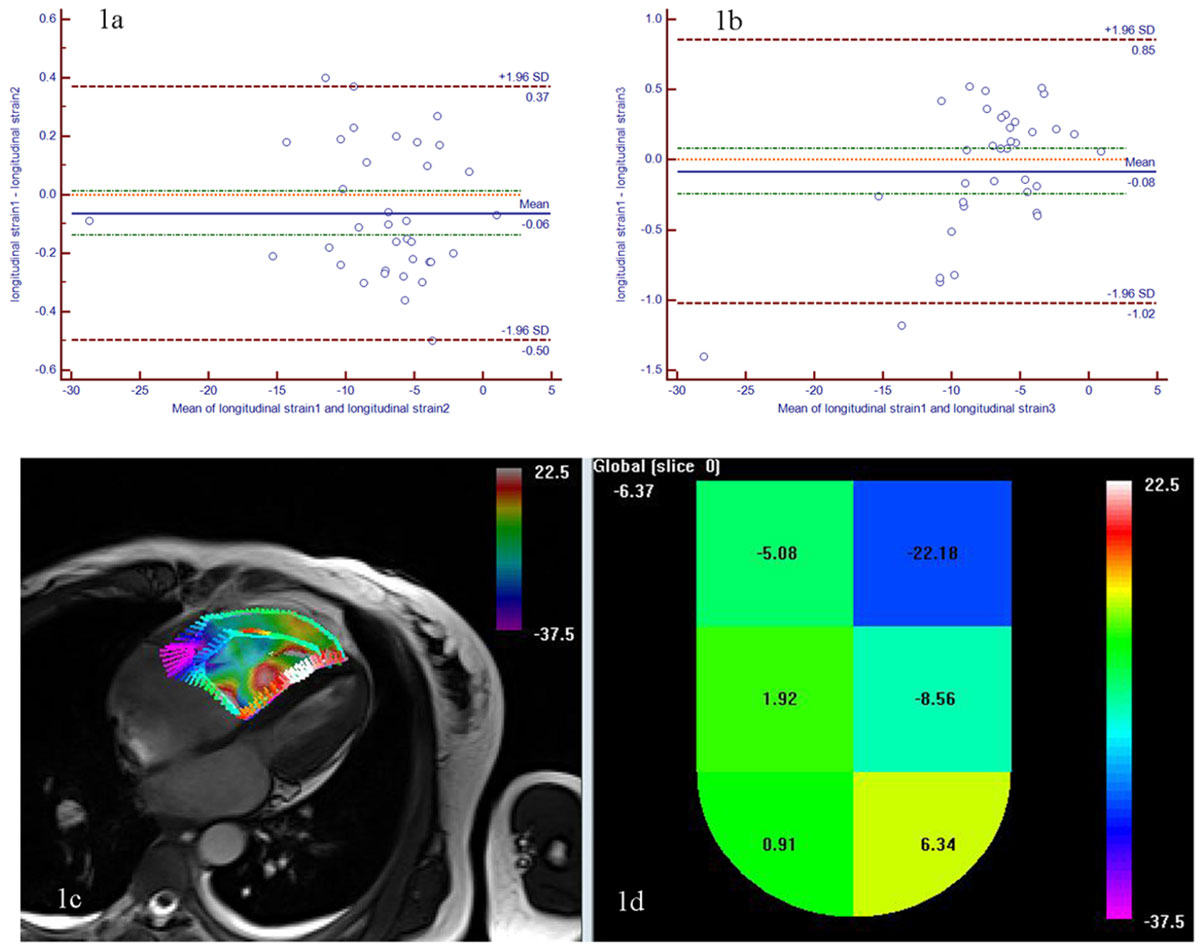
**showed the feasibility of the longitudinal strain was assessed with Trufistrain in twice on Figure 1a and by two readers on Figure 1b**. The longitudinal strain on the four-chamber image on Figre 1c and the segmental and global longitudinal strain on Figure 1d were assessed with Trufistrain

A Bland-Altman plot (Figure 1a) of the longitudinal strain measurements twice showed that the bias was only -0.06 and the interclass correlation coefficient is 0.99 (p < 0.001). A Bland-Altman plot (Figure 1b) of the longitudinal strain measurements by two observers showed that the bias was only -0.08 and the interclass correlation coefficient is 0.96 (p < 0.001). The mean longitudinal strain on 4-chamber cine images (Fig1c and 1d)was -7.20 ± 5.18%, correlating with systolic PAP (r = 0.533,P=0.11), diastolic PAP (r = 0.479,p=0.024), mean PAP (r = 0.575,P=0.005), Cardiac Output (r=-0.500,p=0.018) and pulmonary vascular resistance (r = 0.785,p < 0.001),but did not correlate with PCWP (R=-0.223,P=0.203) and CI (r=-0.355,p=0.105).

## Conclusions

In this work, we demonstrated the feasibility of the longitudinal strain measurement on Cine images by Trufistrain. And the longitudinal strain correlated with PAP, CO and PVR. These suggest longitudinal strain measurement on Cine images by Trufistrain is promising to assess hemodynamics. In the future, we will analyze the correlation of longitudinal strain with myocardial function and fibrosis.

